# Dihydrogeodin from *Fennellia flavipes* Modulates Platelet Aggregation via Downregulation of Calcium Signaling, αIIbβ_3_ Integrins, MAPK, and PI3K/Akt Pathways

**DOI:** 10.3390/md23050212

**Published:** 2025-05-17

**Authors:** Abdul Wahab Akram, Dae-Cheol Choi, Hyung-Kyu Chae, Sung Dae Kim, Dongmi Kwak, Bong-Sik Yun, Man Hee Rhee

**Affiliations:** 1 Department of Veterinary Medicine, College of Veterinary Medicine, Kyungpook National University, Daegu 41566, Republic of Korea; ab.wahab226@gmail.com (A.W.A.);; 2 Division of Biotechnology and Advanced Institute of Environment and Bioscience, College of Environmental and Bioresource Sciences, Jeonbuk National University, Gobong-ro 79, Iksan 54596, Republic of Korea; 3 Institute for Veterinary Biomedical Science, College of Veterinary Medicine Kyungpook National University, Daegu 41566, Republic of Korea

**Keywords:** dihydrogeodin, *Fennellia flavipes*, platelet aggregation, MAPK and PI3K/Akt pathways, marine natural products

## Abstract

Cardiovascular disease remains a leading cause of morbidity and mortality worldwide, frequently arising from platelet hyperactivation and subsequent thrombus formation. Although conventional antiplatelet therapies are available, challenges, such as drug resistance and bleeding complications, require the development of novel agents. In this study, dihydrogeodin (DHG) was isolated from *Fennellia flavipes* and evaluated using platelets derived from Sprague–Dawley rats. Platelet aggregation induced by collagen, adenosine diphosphate, or thrombin was assessed by light transmission aggregometry; DHG significantly reduced aggregation in a dose-dependent manner. Further assays demonstrated that DHG suppressed intracellular calcium mobilization, adenosine triphosphate release, and integrin αIIbβ_3_-dependent fibrinogen binding, thereby impairing clot retraction. Western blot analysis revealed that DHG reduced the phosphorylation of mitogen-activated protein kinases (ERK, JNK, p38) and PI3K/Akt, indicating inhibition across multiple platelet-signaling pathways. Additionally, SwissADME-assisted pharmacokinetics predicted favorable properties without violations of the Lipinski (Pfizer) filter, Muegge (Bayer) filter, Ghose filter, Veber filter, and Egan filter, and network pharmacology revealed inhibition of calcium and MAPK pathways. These results highlight the potential of DHG as a novel antiplatelet agent with broad-spectrum activity and promising drug-like characteristics. Further studies are warranted to assess its therapeutic window, safety profile, and potential for synergistic use with existing antiplatelet drugs.

## 1. Introduction

Cardiovascular disease encompasses a broad spectrum of conditions, including atherosclerosis, coronary heart disease, hypertension, and stroke [1]. A key contributor to cardiovascular disease pathophysiology is platelet hyperactivation, which facilitates atherosclerotic plaque development, plaque rupture, and thrombus formation [2]. Under physiological conditions, platelets play a critical role in maintaining hemostasis [3]; however, when excessively activated, they can form occlusive thrombi that impair blood flow, potentially resulting in myocardial infarction, stroke, or other ischemic complications [4]. Accordingly, pharmacological inhibition of platelet function remains a well-established approach for both the treatment and prevention of cardiovascular disease [5,6,7,8]. While synthetic antiplatelet agents have been instrumental in managing cardiovascular diseases, their use is often accompanied by significant adverse effects. For instance, clopidogrel has been associated with rare but severe hematological disorders, such as aplastic anemia and thrombotic thrombocytopenic purpura. Similarly, long-term aspirin therapy is linked to gastrointestinal complications, including ulcer formation and an increased risk of bleeding [9]. Considering this, the ethnomedical approach could be a promising strategy for preventing cardiovascular disease and its complications [6].

Platelets contain dense (δ) and alpha (α) granules. δ granules store adenosine diphosphate (ADP), adenosine triphosphate (ATP), Ca^2+^, and serotonin, whereas α granules contain fibronectin, fibrinogen, von Willebrand factor (vWF), and P-selectin [6,10]. Multiple signaling pathways control granule secretion, including Src family kinases (early platelet activation), intracellular Ca^2+^ mobilization, the cAMP-VASP-PKA pathway, protein kinase C (PKC) activation, the mitogen-activated protein kinase (MAPK) and phosphoinositide 3-kinase (PI3K)/protein kinase B (Akt) cascades, and RhoA-regulated cytoskeletal reorganization [10]. The increase in intracellular Ca^2+^ is essential for platelet function, initiating downstream signaling that promotes aggregation and thrombus formation [11]. Platelet activation also depends on integrin αIIbβ_3_, which undergoes conformational changes during inside–out signaling and binds fibrinogen, a critical step in the formation of stable thrombi [12]. Fibronectin enhances aggregation by interacting with integrins and recruiting signaling molecules to the cytoplasmic tails [13]. Additionally, Rho GTPases and Rho kinases (ROCKs) regulate cytoskeletal remodeling through myosin light chain phosphorylation, influencing platelet morphology, motility, and clot retraction [14]. Notably, treatment with natural products has been shown to reduce Ca^2+^ mobilization, ATP and serotonin release, fibrinogen binding, fibronectin adhesion, and clot retraction, indicating that such compounds exert antiplatelet effects through the inhibition of granule secretion and integrin αIIbβ_3_ signaling [12,15]. Despite the effectiveness of current antiplatelet agents, such as aspirin and clopidogrel, these drugs are frequently associated with adverse effects and complications [16,17]. Aspirin, for instance, may cause severe gastric ulcers and prolonged bleeding, whereas clopidogrel has been linked to aplastic anemia and thrombocytopenic purpura [6]. Moreover, a substantial proportion of patients exhibit resistance to these therapies [18,19]. Factors contributing to aspirin resistance include genetic polymorphisms in cyclooxygenase-1 (COX-1) and other thromboxane-related genes, upregulated thromboxane biosynthesis from non-platelet sources, elevated platelet turnover, and interindividual variability in drug absorption [5]. These limitations highlight the pressing need for safer and more efficacious antiplatelet agents.

Natural products have historically been a rich source of bioactive molecules [20,21,22]. Numerous plant-derived and microbial metabolites demonstrate potent antiplatelet activity by targeting key enzymes or receptors involved in platelet aggregation [20,23,24,25]. These multitarget mechanisms reflect the broader therapeutic potential of natural compounds, which often exert bidirectional regulatory effects on physiological processes and tend to produce fewer side effects. Several marine fungi have previously been reported to possess bioactive natural products and significantly beneficial antiplatelet activities; for example, *Epicoccum sorghinum* metabolites inhibit platelet aggregation and inflammation [26], and *Aspergillus* species have been reported to exhibit antiplatelet as well as anticancer properties [27]. Moreover, marine fungal lipid bioactives display anti-inflammatory and antithrombotic effects, underscoring their promise as sources of novel antiplatelet agents [28].

Among microbial sources, *Aspergillus* species are well known for producing a wide variety of secondary metabolites with therapeutic potential, including antiviral xanthones, diphenyl ether and butenolide derivatives with α-glucosidase inhibitory activity, and benzophenones with antimicrobial properties [29]. Chlorinated diphenyl ethers, in particular, serve as chemical markers of *Aspergillus* spp. and are generally believed to originate from anthraquinone precursors, such as emodin, via intermediates, such as sulochrin and grisandienes (e.g., geodin) [29]. The genus *Fennellia* (family Trichocomaceae), which includes *F. flavipes* and *F. nivea*, has been identified as the teleomorph of *Aspergillus flavipes* [30]. Although *A. flavipes* has been extensively studied for its production of cytochalasins, alkaloids, polyketides, butenolides, meroterpenoids, diphenyl ethers, benzophenones, and xanthones [31], some of which exhibit cytotoxic, antibacterial, or glucosidase-inhibiting properties, relatively little is known about the bioactive compounds specifically produced by *F. flavipes* [31]. This study began to address this gap by identifying several notable metabolites from *F. flavipes*, including a newly characterized dimeric 1,3-dihydroisobenzofuran and five previously known compounds, such as dihydrogeodin (DHG) [31,32]. In the present study, we isolated DHG from the culture broth of *F. flavipes* and evaluated its in vitro antiplatelet activity. Our findings demonstrate that DHG functions as a potent inhibitor of platelet aggregation, highlighting the potential of *F. flavipes* as a source of novel antiplatelet agents and pharmacologically active natural products.

## 2. Results

### 2.1. Structural Elucidation

The known compound dihydrogeodin was identified through the comprehensive analysis of its ^1^H and ^13^C NMR spectra and HPLC data (Supplementary Figures S1–S5), which were consistent with both recent [31] and previously published reports [32,33].

### 2.2. DHG Inhibits Agonist-Induced Platelet Aggregation

**Figure 1:**
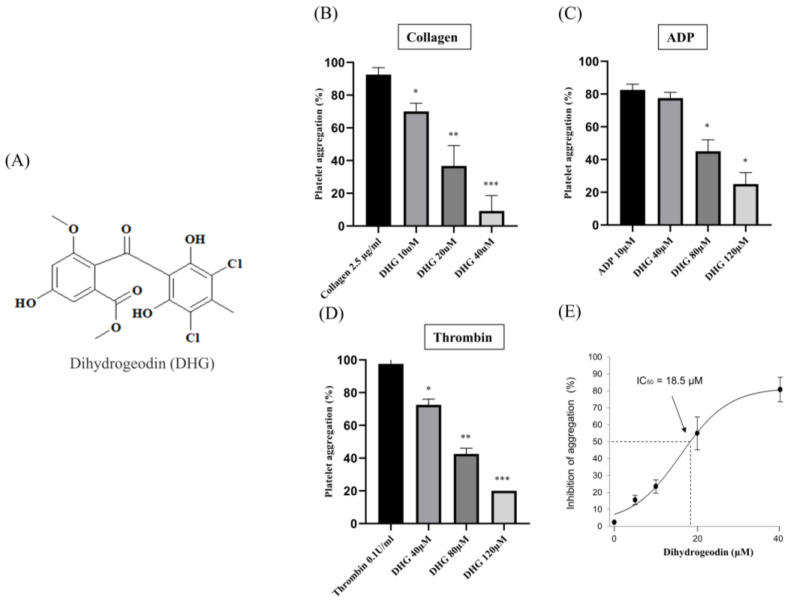
Dihydrogeodin (DHG) inhibits agonist-induced platelet aggregation. Washed platelets were incubated for 1 min with DHG in the presence of 1 mM CaCl_2_. Aggregation was initiated by the addition of agonists. (**A**) Chemical structure of DHG. (**B**) Effect of DHG on collagen-induced platelet aggregation. (**C**) Effect of DHG on ADP-induced platelet aggregation. (**D**) Effect of DHG on thrombin-induced platelet aggregation. (**E**) IC_50_ of DHG for collagen-induced platelet aggregation. Data are expressed as means ± SD (*n* = 3). Statistical significance was determined by one-way ANOVA followed by a post hoc Dunnett’s test. * *p* < 0.05, ** *p* < 0.01, *** *p* < 0.001 versus agonist.

Platelet aggregation can be induced by various agonists, each activating distinct signaling pathways. Collagen initiates platelet adhesion and aggregation at sites of vascular injury [34], ADP promotes platelet activation through P_2_Y receptors [35], and thrombin induces robust platelet responses via protease-activated receptors [36]. To assess the antiplatelet potential of DHG, we evaluated platelet aggregation induced by each of these three agonists. Figure 1A presents the chemical structure of DHG. As shown in Figure 1B–D, DHG significantly inhibited platelet aggregation induced by collagen, ADP, and thrombin in a dose-dependent manner. Notably, DHG exerted its strongest inhibitory effect on collagen-induced aggregation. In collagen-stimulated platelets (Figure 1B), DHG at 10 µM significantly reduced aggregation compared with collagen alone (* *p* < 0.05) and completely inhibited aggregation at 40 µM (*** *p* < 0.001). In contrast, ADP-induced aggregation (Figure 1C) required a concentration of 80 μM to achieve significant reduction (* *p* < 0.05), whereas thrombin-induced aggregation (Figure 1D) was significantly inhibited at 40 µM (* *p* < 0.05). These findings indicate that DHG more strongly targets the collagen-mediated pathway of platelet activation, while also exhibiting broader inhibitory effects against ADP- and thrombin-induced activation, highlighting its potential as a novel therapeutic agent for the prevention of platelet-mediated thrombotic events. The IC_50_ of DHG for collagen-induced platelet aggregation was calculated to be 18.5 µM (Figure 1E). No potential cytotoxicity was observed at the indicated concentration (Supplementary Figure S7).

### 2.3. DHG Inhibits ATP Release and [Ca^2+^]_i_ Mobilization

**Figure 2:**
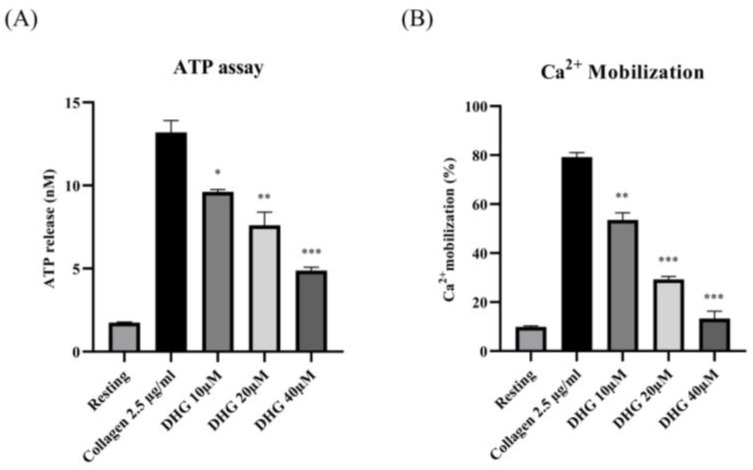
DHG inhibits platelet granule release. (**A**) Platelets were preincubated with DHG, stimulated with collagen, and the supernatant was analyzed for ATP using a standard ATP assay kit. (**B**) Intracellular Ca^2+^ levels were assessed using Fura-2/AM-loaded platelets. Data are presented as means ± SD (*n* = 3). * *p* < 0.05, ** *p* < 0.01, *** *p* < 0.001 versus collagen alone.

To determine whether DHG affects dense granule secretion, we measured ATP release from platelets stimulated with collagen (2.5 µg/mL). As shown in Figure 2 (left panel), collagen substantially increased ATP release compared with resting platelets, whereas DHG at 10, 20, and 40 μM significantly reduced ATP secretion in a dose-dependent manner (* *p* < 0.05, ** *p* < 0.01, *** *p* < 0.001 versus collagen-stimulated controls). In parallel, intracellular Ca^2+^ mobilization, a key event in platelet activation, was assessed using Fura-2/AM-loaded platelets. Collagen stimulation induced a substantial increase in Ca^2+^ levels relative to resting platelets (Figure 2, right panel). DHG at 10, 20, and 40 μM significantly attenuated this collagen-induced Ca^2+^ mobilization (** *p* < 0.01, *** *p* < 0.001 versus collagen-stimulated controls). These findings suggest that DHG suppresses both dense granule secretion and intracellular calcium signaling, providing further mechanistic evidence for its potent antiplatelet activity.

### 2.4. DHG Downregulates Inside–Out and Outside–In Signaling and Prevents Platelet Shape Change

**Figure 3:**
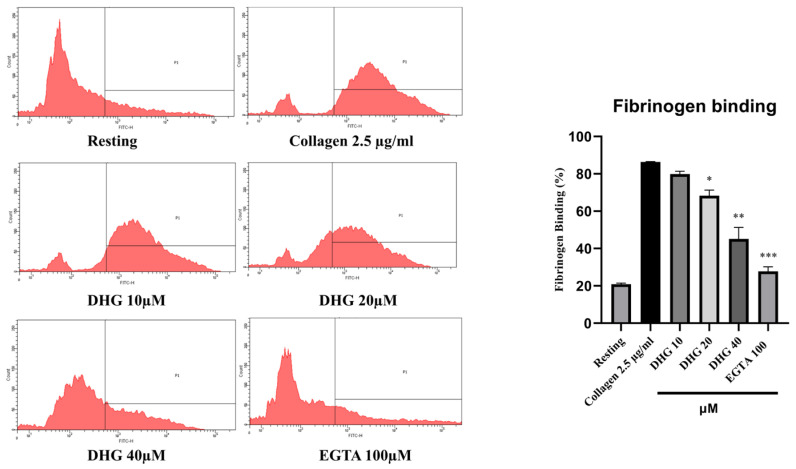
DHG inhibits integrin αIIbβ3-mediated inside–out signaling pathway. (**Left**) Flow cytometry histograms showing fibrinogen binding in resting and collagen-stimulated platelets (2.5 μg/mL) in the presence or absence of DHG (10, 20, 40 μM) or EGTA (100 μM). (**Right**) Quantification of fibrinogen binding. Data are presented as means ± SD (*n* = 3). * *p* < 0.05, ** *p* < 0.01, *** *p* < 0.001 versus collagen alone.

Inside–out signaling in platelets is primarily mediated by the activation of integrin αIIbβ_3_, which promotes fibrinogen binding and supports platelet aggregation [37]. As shown in Figure 3 (left), collagen stimulation (2.5 µg/mL) substantially increased the proportion of platelets binding fibrinogen compared with resting conditions. Pretreatment with DHG at 10, 20, and 40 μM significantly reduced fibrinogen binding in a dose-dependent manner (* *p* < 0.05, ** *p* < 0.01, *** *p* < 0.001 versus collagen-stimulated controls). Notably, DHG at 40 μM nearly abolished fibrinogen binding, producing an effect comparable to that of EGTA (100 μM), which chelates calcium and prevents integrin activation [38]. These results indicate that DHG potently inhibits the inside–out signaling pathway leading to αIIbβ_3_ activation, thereby blocking fibrinogen binding and platelet aggregation.

**Figure 4:**
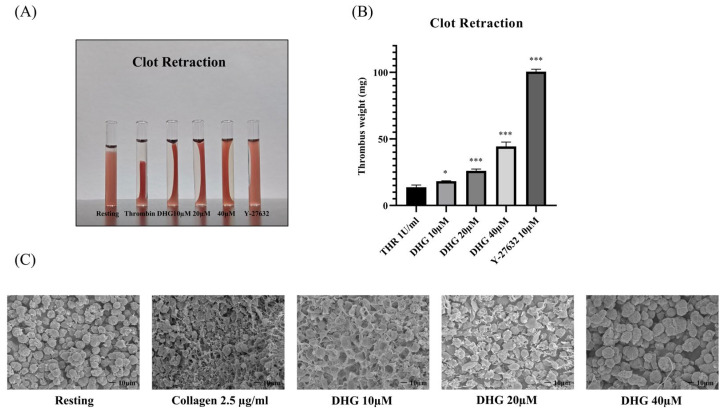
DHG inhibits clot retraction. Platelet-rich plasma (PRP) was incubated for 2 min with DHG or Y-27632. Human thrombin (1 U/mL) was then added, and (**A**) clot retraction and clot shape change were assessed at room temperature for 90 min. (**B**) The clot weight was assessed * *p* < 0.05, *** *p* < 0.001 versus thrombin alone. (**C**) Platelet shape change was visualized with scanning electron microscopy (SEM). SEM images of the platelet shape revealed a change in morphology from discoid to a rounded shape containing filopodia upon activation with collagen, which was prevented by treatment with DHG.

After integrin activation and aggregation, outside–in signaling via αIIbβ_3_ coordinates platelet spreading, cytoskeletal reorganization, and clot retraction, a critical process for thrombus stabilization [12]. To assess whether DHG interferes with outside–in signaling, we performed a clot retraction assay using thrombin (1 U/mL) as the stimulus (Figure 4). In the absence of DHG, clot retraction was substantial, resulting in a compact thrombus (Figure 4A). In contrast, treatment with DHG at 20 and 40 μM significantly impaired clot retraction (* *p* < 0.05, ** *p* < 0.01, *** *p* < 0.001 versus thrombin alone), yielding larger, less compact, and heavier clots (Figure 4B). The degree of inhibition was comparable to that observed with Y-27632 (10 μM), a known ROCK inhibitor, suggesting that DHG interferes with Rho-dependent signaling pathways. These findings demonstrate that DHG disrupts both inside–out and outside–in signaling in platelets. Further scanning electron microscopy (SEM) was performed to visualize the platelet shape change (Figure 4C). Collagen-induced platelets show clear fibrin meshwork formation which was significantly prevented after treatment with the indicated concentration of DHG.

### 2.5. DHG Attenuates MAPK and PI3K/Akt Phosphorylation

**Figure 5:**
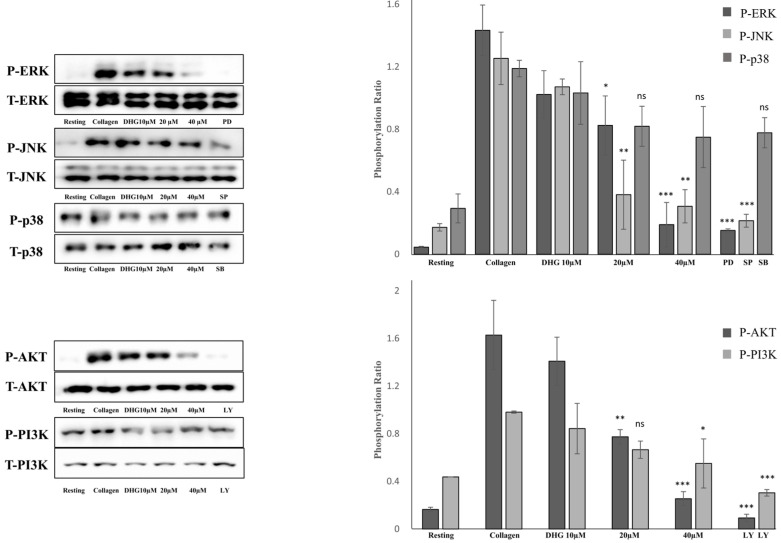
DHG inhibits protein phosphorylation. Platelets were preincubated with DHG and stimulated with collagen. Aggregation was halted by the addition of lysis buffer, after which protein concentrations were measured. Proteins were separated by SDS-PAGE, transferred to PVDF membranes, and probed with specific antibodies after membrane blocking. Visualization was performed using enhanced chemiluminescence. Representative Western blots (**left panels**) and corresponding quantification (**right panels**) show the phosphorylation levels of MAPK (ERK, JNK, p38) and PI3K/Akt in platelets pretreated with DHG (10, 20, 40 μM) or a reference inhibitor. Data are presented as means ± SD. Statistical significance was determined by one-way ANOVA followed by a post hoc Dunnett’s test * *p* < 0.05, ** *p* < 0.01, *** *p* < 0.001, and ns = non-significant versus collagen alone.

To determine whether DHG modulates the MAPK and PI3K/Akt pathways, both of which are essential for platelet activation [5], platelets were pretreated with DHG (10, 20, or 40 μM) and subsequently stimulated with collagen (2.5 µg/mL). Phosphorylation levels of extracellular signal-regulated kinase (ERK), Janus kinase (JNK), p38, PI3K, and Akt were assessed by Western blotting (Figure 5). Collagen stimulation substantially increased the phosphorylation of these proteins compared with resting platelets. In contrast, DHG reduced phosphorylation in a dose-dependent manner, whereas total protein levels (T-ERK, T-JNK, T-p38, T-PI3K, T-Akt) remained largely unchanged. Notably, DHG at 40 µM reduced phospho-ERK, phospho-JNK, phospho-PI3K, and phospho-Akt levels to near-basal values, indicating a strong inhibitory effect on MAPK and PI3K/Akt signaling. These results suggest that suppression of these key pathways contributes to the antiplatelet mechanism of DHG.

### 2.6. Pharmacokinetics, Drug-Likeliness Analysis

**Figure 6:**
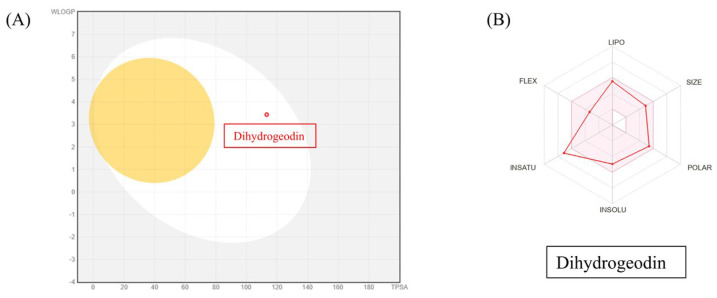
SwissADME analysis of DHG. The canonical SMILES for DHG were retrieved from PubChem and entered into SwissADME. Output files and visualizations were directly from the website. (**A**) The BOILED-Egg model evaluated human intestinal absorption (HIA) and blood–brain barrier (BBB) permeability based on lipophilicity and polarity, generating a WLOGP versus tPSA plot. (**B**) SwissADME predictions showed that DHG complies with Lipinski’s rule of five, indicating good oral bioavailability.

To assess the drug-likeness and pharmacokinetic potential of DHG, we utilized SwissADME to predict its physicochemical and pharmacokinetic properties. The analysis revealed that DHG complies with Lipinski’s rule of five, suggesting good oral bioavailability. Additionally, DHG demonstrated favorable lipophilicity (LogP), solubility, and gastrointestinal (GI) absorption, supporting its potential for systemic administration. No violations of drug-likeness criteria were predicted, reinforcing its suitability as a bioactive compound. The pharmacokinetic profile also suggested a low probability of BBB permeability, which may reduce the risk of central nervous system side effects. DHG was also predicted to be a non-substrate for P-glycoprotein (P-gp) efflux transporters, potentially enhancing its bioavailability. However, predicted interactions with cytochrome P450 (CYP) isoforms warrant consideration in future metabolism studies (Figure 6A,B).

### 2.7. Network Pharmacology

**Figure 7:**
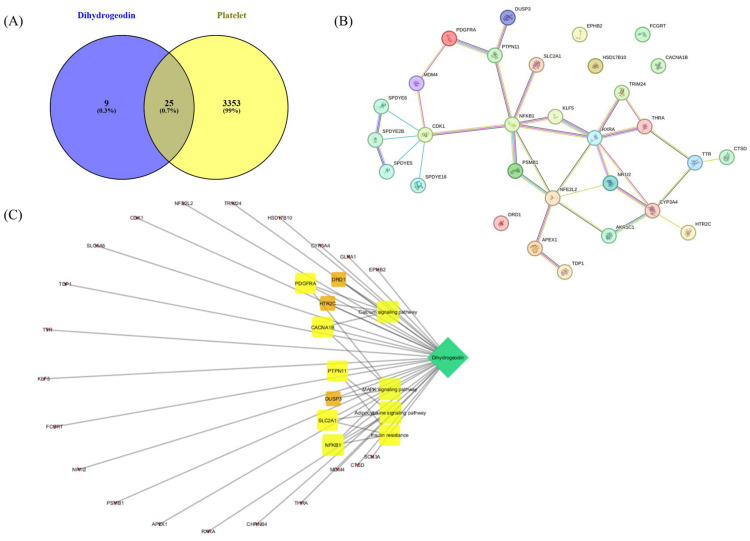
Network pharmacological assessment of DHG. (**A**) Venn diagram showing the intersection between predicted targets of DHG and platelet-related genes, where 25 overlapping genes were identified. (**B**) Protein–protein interaction (PPI) network of the 25 common targets constructed using the STRING database. (**C**) Compound–target–pathway network illustrating the association between DHG, its core targets, and enriched KEGG pathways.

To explore the potential mechanism of DHG in platelet modulation, a network pharmacological approach was employed. A Venn diagram (Figure 7A) was constructed to identify overlapping targets between DHG and platelet activation-related genes, revealing 25 common targets, suggesting a potential pharmacological link between the compound and platelet function. A protein–protein interaction (PPI) network of these overlapping targets was constructed using the STRING database (Figure 7B). Notably, NFKB1, PDGFRA, RXRA, and PTPN11 emerged as key hub nodes, indicating their potential central roles in mediating the effects of DHG. The network shows that these targets are functionally interconnected and may be critical in signal transduction processes related to platelet activity. Further, a compound–target–pathway network (Figure 7C) demonstrated that DHG targets are significantly enriched in several signaling pathways. These include the calcium signaling pathway, MAPK signaling pathway, adipocytokine signaling pathway, and insulin resistance pathways. Notably, these pathways are not only relevant to cardiovascular diseases but also play crucial roles in platelet activation and aggregation, highlighting the therapeutic potential of DHG in thrombosis or related disorders.

## 3. Discussion

Natural products have attracted considerable interest due to their multitarget activities and relatively low incidence of adverse effects [39]. Recent studies have identified marine-derived compounds as promising sources of antiplatelet agents [40,41,42,43]. Similarly, various herbal medicines and their purified constituents have demonstrated potent antiplatelet activity [6,44,45]. For example, in our recently published study, ginsenoside Rg5 regulates GPVI signaling pathways by inhibiting collagen-induced platelet aggregation. The cryptotanshinone from *Salvia miltiorrhiza* Bunge exerts P2Y_12_-independent antiplatelet effects by downregulating the PI3K/Akt, MAPK, and STAT3 pathways [46]. Tryptanthrin from *Isatidis Radix* interferes with both inside–out and outside–in αIIbβ_3_ signaling, thus preventing stable thrombus formation [45]. Additionally, dieckol from *Eisenia bicyclis* inhibits platelet aggregation by suppressing granule secretion, integrin αIIbβ_3_ function, and the RhoA/ROCK pathway [12]. Similarly, in our study, DHG attenuated integrin αIIbβ_3_–mediated fibrinogen binding and clot retraction, as well as the phosphorylation of MAPK (ERK, JNK, p38) and PI3K/Akt, underscoring its multifaceted mode of action.

Platelet activation depends on a coordinated network of signaling events triggered by vascular injury [47]. Collagen, ADP, and thrombin activate distinct platelet receptors, GPVI, P2Y_12_, and protease-activated receptors, respectively, to initiate granule secretion, integrin αIIbβ_3_ activation, and cytoskeletal reorganization [48]. DHG significantly inhibited platelet aggregation induced by collagen, ADP, and thrombin in a dose-dependent manner, indicating broad inhibitory activity across multiple platelet activation pathways (Figure 1). In addition to its inhibition of aggregation, DHG substantially reduced intracellular Ca^2+^ mobilization and ATP release in response to collagen stimulation (Figure 2). Given the critical role of Ca^2+^ in platelet granule secretion, these results suggest that DHG disrupts upstream signaling events essential for platelet activation and may prevent thrombus formation at an early stage. DHG significantly reduced fibrinogen binding to platelets, indicating strong inhibition of αIIbβ_3_ activation. By blocking this step, DHG prevents the formation of stable platelet plugs (Figure 3). Furthermore, DHG attenuated human thrombin-induced clot retraction, mirroring the effects of established ROCK inhibitors and also prevented collagen-induced platelet shape change (Figure 4). DHG also reduced the phosphorylation of key signaling proteins in a dose-dependent manner without significantly altering total protein levels (Figure 5). Additionally, DHG satisfies Lipinski’s rule of five, suggesting good oral bioavailability. It also demonstrated favorable lipophilicity, solubility, and gastrointestinal absorption, along with a low probability of blood–brain barrier permeability (Figure 6A,B). The Venn diagram in Figure 7A shows the intersection between DHG-predicted targets and genes associated with platelet activation. Among the 34 DHG-related genes and 3353 platelet activation-related genes, 25 common genes were identified, representing potential targets through which DHG may influence platelet function. Figure 7B presents a protein–protein interaction (PPI) network constructed from the 25 overlapping genes using the STRING database. The network reveals a tightly connected hub centered around key nodes, such as NFKB1, CDK1, RXRA, PDGFRA, and PTPN11, indicating their crucial roles in mediating the interaction between DHG and platelet-related signaling. These core targets are functionally linked through multiple biological processes, suggesting DHG may exert multi-targeted modulation. Further, Figure 7C shows the compound–target–pathway network constructed using Cytoscape. DHG is connected to several targets, including NFKB1, PDGFRA, DRD1, PTPN11, and HTR2C, which are further linked to relevant pathways, such as the MAPK signaling pathway, calcium signaling pathway, adipocytokine signaling pathway, and insulin resistance. These enriched pathways have established roles in platelet function, inflammation, and cardiovascular regulation, supporting the potential of DHG to modulate platelet activity through multiple signaling cascades. Taken together, these pharmacological properties support DHG as a promising candidate for antithrombotic drug development.

**Figure 8:**
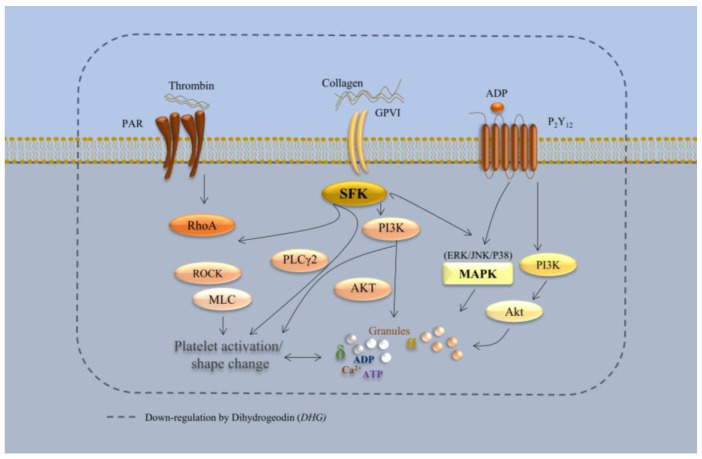
Schematic representation of DHG-mediated inhibition of platelet signaling pathways. DHG inhibits platelet aggregation while reducing ATP release, intracellular Ca^2+^ mobilization, αIIbβ3 integrin activation, clot retraction, and activation of the MAPK and PI3K/Akt pathways.

Unlike many conventional antiplatelet agents, which typically target a single receptor or enzyme (e.g., P2Y_12_ for clopidogrel or COX-1 for aspirin), DHG appears to modulate multiple pathways integral to platelet function. Our results demonstrate that DHG inhibits platelet aggregation while reducing ATP release, intracellular Ca^2+^ mobilization, αIIbβ_3_ integrin activation, clot retraction, and activation of the MAPK and PI3K/Akt pathways (see schematic in Figure 8). Collectively, these findings suggest a broad-spectrum antiplatelet mechanism, similar to that of other recently reported multitarget natural compounds, such as ginsenoside Rg5 [15] cryptotanshinone [46], dieckol [12], and tryptanthrin [45], which also inhibit platelet activation by modulating multiple signaling cascades. However, in vivo studies are necessary to further elucidate the pharmacological effects of DHG in animal models or human platelets. Although the in vitro findings strongly support its antiplatelet efficacy, translation of these results to in vivo systems requires additional investigation. Therefore, future studies using animal models of arterial thrombosis (e.g., FeCl_3_-induced vascular injury) or venous thrombosis are essential to establish its therapeutic potential.

## 4. Materials and Methods

### 4.1. Reagents

Collagen, ADP, and thrombin were obtained from Chrono-Log Co. (Geffen, The Netherlands). Human plasma-derived thrombin and glutaraldehyde were purchased from Sigma-Aldrich. ATP assay kits were acquired from Cayman Chemical (Ann Arbor, MI, USA). Paraformaldehyde, Fura 2-AM, and Alexa Fluor 488-conjugated fibrinogen were obtained from Invitrogen (Eugene, OR, USA). Avertin was prepared as the anesthetic by dissolving 2,2,2-tribromoethanol (Sigma-Aldrich, Darmstadt, Germany, catalog no. T48402-5G) in 2-methyl-2-butanol (Sigma-Aldrich, Darmstadt, Germany, catalog no. 152463). All antibodies for western blotting were purchased from Cell Signaling Technology (Danvers, MA, USA).

### 4.2. Fungal Material and Fermentation

A fungal strain of *F. flavipes* (FU00000759) was obtained from the National Marine Biodiversity Institute of Korea (MABIK) (Seocheon-gun, Chungcheongnam-do 33662). This strain was isolated from a tidal flat in Incheon, located on the Western Coast of Korea. The strain was cultured in twenty 5 L flasks, each containing 1 L of potato dextrose broth, at 27 °C for 4 weeks under stationary conditions.

### 4.3. Extraction, Isolation, and Structure Determination

The extraction, isolation, and structure determination of the active compounds from *F. flavipes* were performed as described in our recent publication [31] and Supplementary Material Section 1.1. The ^1^H and ^13^C NMR spectra matched those known for dihydrogeodin [31,32], and complete data are provided in Supplementary Material Figures S1–S5.

### 4.4. Experimental Animals

Seven-week-old male Sprague–Dawley rats weighing 240–260 g were used for in vitro platelet experiments. The animals were acclimated in an environmentally controlled room maintained at approximately 23 ± 2 °C and 50% ± 10% humidity, with a 12 h light/dark cycle. All animal procedures were performed in accordance with institutional guidelines and approved by the Animal Care Committee of the College of Veterinary Medicine, Kyungpook National University, Daegu, Republic of Korea (Permit no KNU 2022-0083).

### 4.5. Light Transmission Aggregometry and Scanning Electron Microscopy (SEM)

Blood was collected from Sprague–Dawley rats via cardiac puncture using a syringe containing acid-citrate-dextrose to isolate purified platelets [6]. The collected blood was transferred into round-bottom tubes containing Tyrode’s buffer and acid-citrate-dextrose at a 1:4 (*v*/*v*) ratio. Washed platelets were separated from whole blood by centrifugation at 170× *g* for 7 min, followed by a second centrifugation at 350× *g* for 10 min. The platelet suspension was adjusted to a concentration of 3 × 10^8^ cells/mL for aggregometry analysis. Platelets were incubated for 1 min with DHG (10, 20, or 40 μM) or with DMSO as a control in the presence of 1 mM CaCl_2_. Aggregation was then induced by the addition of agonists (collagen at 2.5 μg/mL, ADP at 10 μM, or thrombin at 0.1 U/mL) for 5 min. This method enabled precise quantification of the platelet response under defined experimental conditions, providing valuable insights into the effects of dihydrogeodin on platelet function. Platelets incubated with DHG and agonist (collagen) were fixed in 0.5% paraformaldehyde and 0.5% osmium tetroxide, dehydrated, and freeze-dried at –55 °C before being mounted for field-emission scanning electron microscopy (Hitachi SU8220, Tokyo, Japan) to assess ultrastructural shape changes.

### 4.6. ATP Release Assay, [Ca^2+^]_i_ Mobilization Assay, and Fibrinogen Binding Assay

Platelets were preincubated with dihydrogeodin and then stimulated with collagen to induce activation. Subsequently, ATP release, intracellular calcium mobilization, and fibrinogen binding were assessed as previously described [6]. ATP secretion was measured in the supernatant using an ATP assay kit to evaluate platelet function. Intracellular calcium concentration ([Ca^2+^]_i_) was determined in Fura-2/AM-loaded platelets using the following formula: 224 nM × (F − F_min_)/(F_max_ − F). For the fibrinogen binding assay, platelets were stained with an anti-fibrinogen antibody, and activation levels were evaluated by flow cytometry after collagen stimulation in the presence or absence of dihydrogeodin.

### 4.7. Clot Retraction

Platelet-rich plasma (PRP, 250 μL) was incubated for 2 min with DHG or Y-27632 (a ROCK inhibitor). A final volume of 1 mL was achieved by adding red blood cells (5 μL) and Tyrode’s buffer. Thrombin (1 U/mL) was then added, and clot retraction was assessed at room temperature. Thrombin-induced clots were weighed to compare clot retraction among treatment groups.

### 4.8. Western Blotting

After preincubation with DHG and stimulation with collagen, lysis buffer was added to initiate lysis, and protein concentrations were quantified. Whole platelet proteins were isolated for subsequent analysis. Proteins were separated by sodium dodecyl sulfate–polyacrylamide gel electrophoresis (SDS-PAGE) and transferred onto polyvinylidene fluoride (PVDF) membranes. Membranes were blocked and incubated overnight with primary antibodies, followed by incubation with secondary antibodies for 3 h. The membranes were then washed three times and visualized using enhanced chemiluminescence. This procedure enabled evaluation of changes in protein expression induced by dihydrogeodin treatment.

### 4.9. Pharmacokinetics, Drug-Likeliness Analysis, and Network Pharmacology

SwissADME, a freely accessible web-based tool, was used to evaluate the pharmacokinetic properties and drug-likeness of DHG, as previously reported [49]. Briefly, the canonical SMILES notation for DHG was retrieved from PubChem (PC-ID 612831 https://pubchem.ncbi.nlm.nih.gov/compound/Dihydrogeodin, accessed on 20 March 2025) and entered into the SwissADME platform (http://www.swissadme.ch/, accessed on 20 March 2025). The resulting output files and images were directly imported from the website. The BOILED-Egg (Brain or Intestinal Estimated permeation predictive) model provides a rapid and straightforward method for assessing human intestinal absorption (HIA) and blood–brain barrier (BBB) permeation by calculating the lipophilicity and polarity of molecules, followed by generation of a water partition coefficient (WLOGP) versus topological polar surface area (tPSA) plot. For network pharmacology, the potential target proteins of DHG were predicted using the SuperPred web server, and the results were downloaded as CSV files. Corresponding UniProt IDs for Homo sapiens were retrieved and used for protein mapping via the STRING database to identify protein names (preferred names) associated with DHG. To identify disease-related targets, the keyword “platelet activation” was searched in the GeneCards database, and relevant human gene targets were extracted. The predicted DHG target genes (compound-related genes) and platelet activation-related genes (disease target genes) were compared using Venny 2.1. This analysis identified overlapping targets (common genes) between DHG and platelet activation. These overlapping targets were input into the STRING database (organism: Homo sapiens) to generate a protein–protein interaction (PPI) network and assess potential biological connections. KEGG pathway enrichment analysis was also performed through STRING to identify key signaling pathways associated with the overlapping genes. Finally, the network of DHG, overlapping target genes, and enriched pathways was constructed and visualized using Cytoscape software (version 3.7.2).

### 4.10. Statistical Analysis

Data were analyzed by one-way analysis of variance (ANOVA), followed by post hoc Dunnett’s test (SAS Institute, Inc., Cary, NC, USA) to determine statistical significance. Results are presented as means ± standard deviation (SD). A *p*-value of 0.05 or less was considered statistically significant.

## 5. Conclusions

Overall, the findings of this study position dihydrogeodin as a promising natural antiplatelet agent capable of multifaceted inhibition of platelet function. By targeting central signaling pathways (MAPK and PI3K/Akt) and integrin αIIbβ_3_ activation, DHG interferes with both the early and late stages of platelet activation. Coupled with its favorable in silico drug-likeness profile, these results support DHG as a strong candidate for future pharmacological development. Rigorous in vivo studies and detailed mechanistic investigations will be required to fully characterize its therapeutic efficacy and safety in the prevention or treatment of thrombotic diseases.

## Data Availability

The original contributions presented in the study are included in the article/Supplementary material; further inquiries can be directed to the corresponding authors.

## Supplementary Materials

The following supporting information can be downloaded at: https://www.mdpi.com/article/10.3390/md23050212/s1, Figure S1. Compound 1, Figure S2. ^1^H NMR spectrum of compound 1, Figure S3. ^13^C NMR spectrum of compound 1, Figure S4. High-resolution ESI-mass spectrometry of compound 1, Figure S5. HPLC analysis of compound 1, Figure S6. Western blot bands, Figure S7. Cytotoxicity of DHG.
