# Supplementary material for: Dihydrogeodin from Fennellia flavipes Modulates Platelet Aggregation via Downregulation of Calcium Signaling, αIIbβ3 Integrins, MAPK, and PI3K/Akt Pathways
Source: Mar Drugs. 2025 May 17;23(5):212. doi: 10.3390/md23050212 (PMC12112792; doi:10.3390/md23050212)
Supplement: Supplementary file 1 [file marinedrugs-23-00212-s001.zip › marinedrugs-3599379-supplementary.pdf]

Supplementary Materials

Dihydrogeodin from *Fennellia flavipes* modulates platelet aggregation via downregulation of calcium signaling, αIIbβ3 integrins, MAPK, and PI3K/Akt pathways

Abdul Wahab Akram [a], Dae-Cheol Choi [b], Hyung-Kyu Chae [a], Sung Dae Kim [a], Dongmi Kwak [a], Bong-Sik Yun [b], *, Man Hee Rhee [a, c] *

1. Supplementary Material

1.1 Isolation and purification

The culture broth of *F. flavipes* (20 L) was filtered with cheese cloth to separate broth filtrate and mycelium. The broth filtrate was partitioned with ethyl acetate, and the mycelium was extracted with acetone for 24 hours at room temperature. The acetone extract was filtered and evaporated under reduced pressure to eliminate acetone, and then, the residue was partitioned between ethyl acetate and water. The ethyl acetate extracts (31.2 g) were combined and subjected to silica gel column chromatography eluted with chloroform:methanol (20:1, 10:1, 5:1, 2:1, 1:1, v/v). The chloroform:methanol (20:1) fraction (1.6 g) was concentrated and then subjected to ODS column chromatography eluted with 30% and 70% aq. methanol, consecutively. The 70% aq. methanol fraction was subjected to reversed-phase MPLC and eluted with a gradient of increasing methanol in water (30→80% aq. methanol), followed by Sephadex LH-20 column chromatography eluted with methanol to give compound **1** (46.9mg).

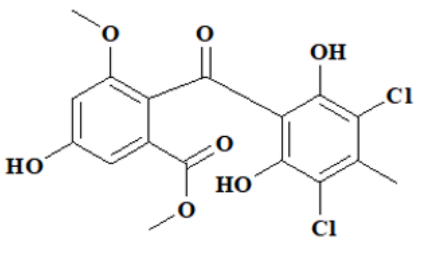

Figure S1. Compound 1.

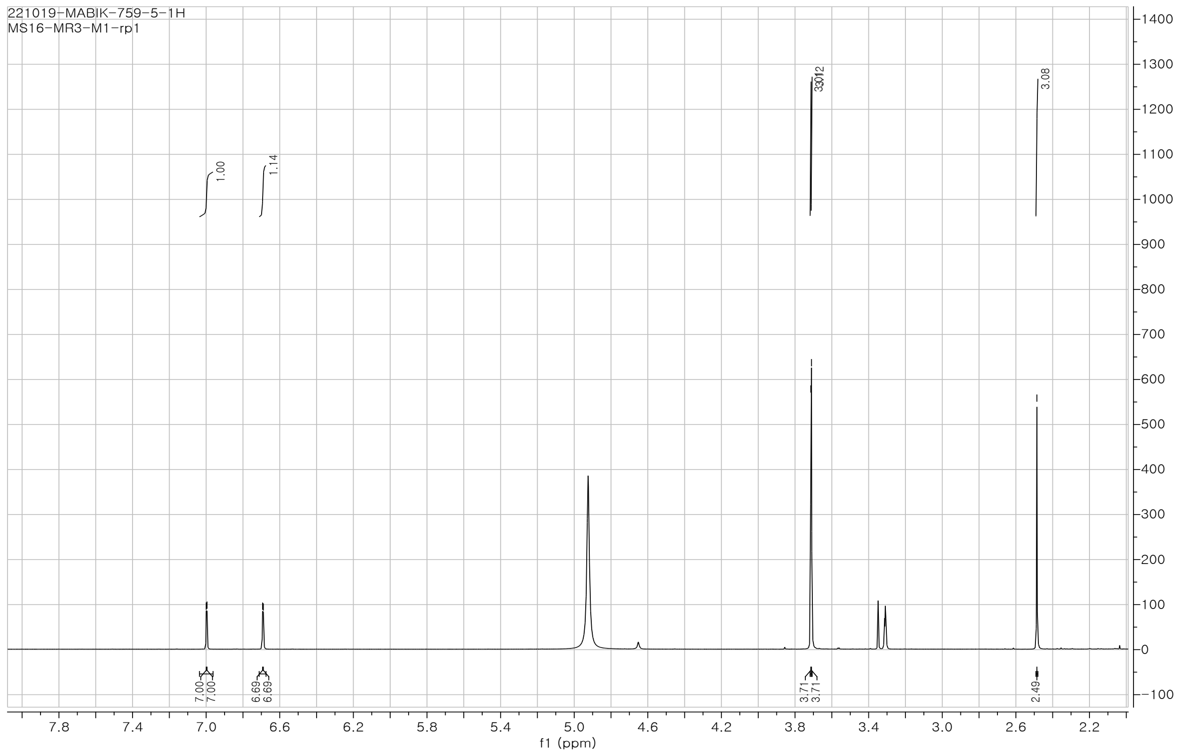

Figure S2. ^{1}H NMR spectrum of compound **1**.

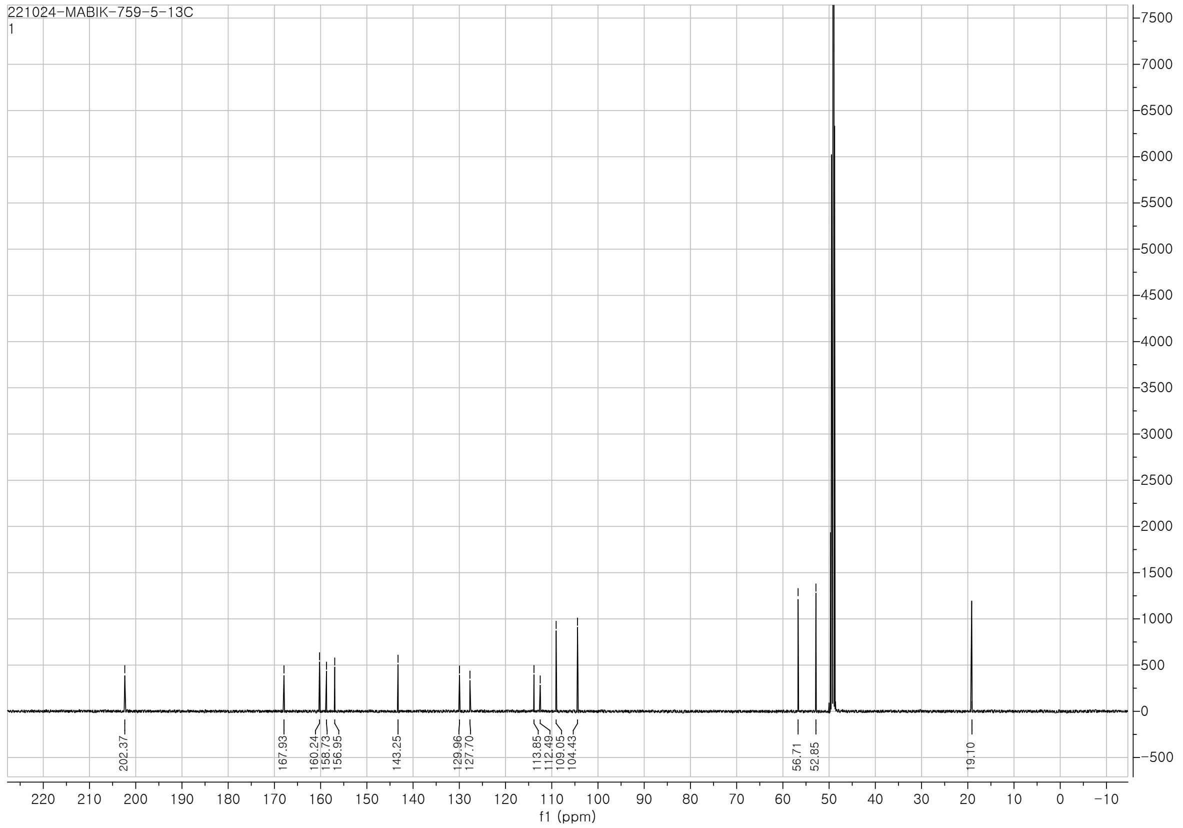

Figure S3. ^{13}C NMR spectrum of compound **1**.

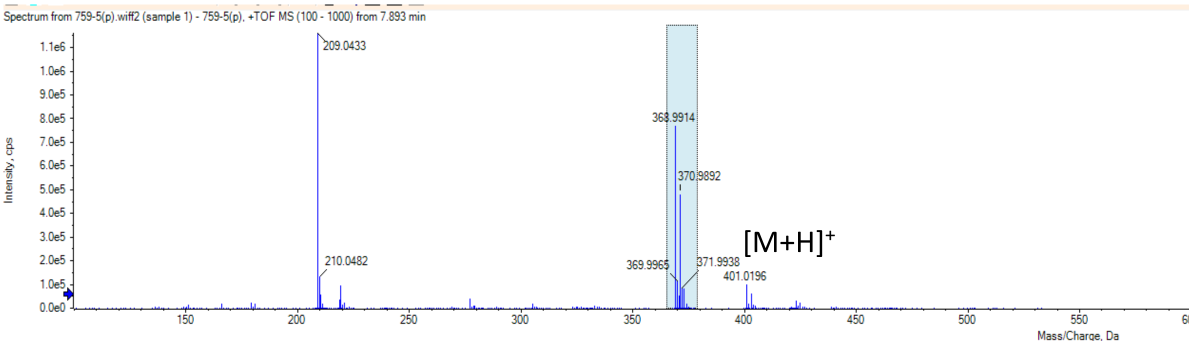

Figure S4. High-resolution ESI-mass spectrometry of compound **1**.

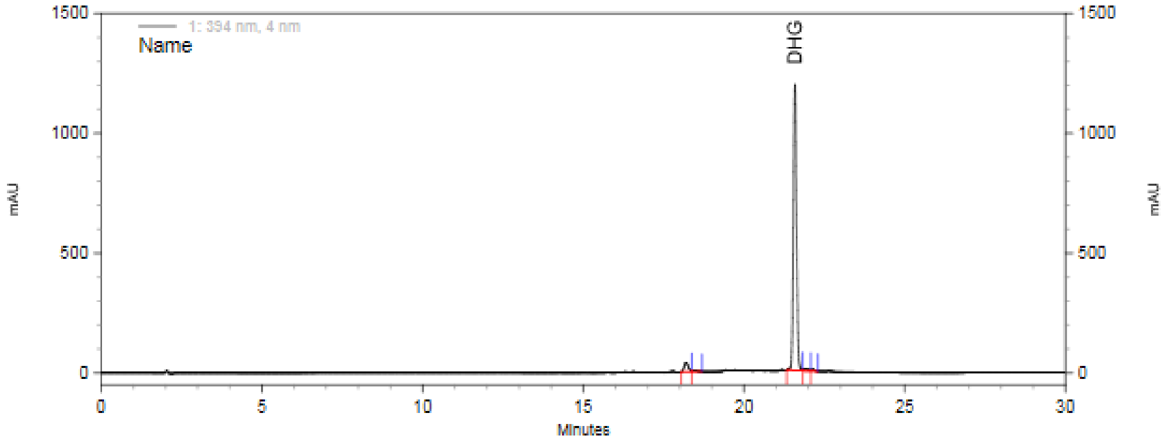

Name	Retention Time	Area Percent
DHG	21.580	93.548

Figure S5. HPLC analysis of compound **1**.

1.2 Western blot

Western blot results demonstrated significant inhibition of the phosphorylation of MAPK and PI3K/Akt with different doses of DHG. The full-length blots for the gel images can be presented as supplementary figure S6a= P-ERK pathway, S6b= P-p38 pathway, S6c= P-JNK pathway, S6d= P-Akt pathway, S6e= PI3K pathway in platelets.

Supplementary Figure S6a

P-ERK (42-44 kDa) **T-ERK**

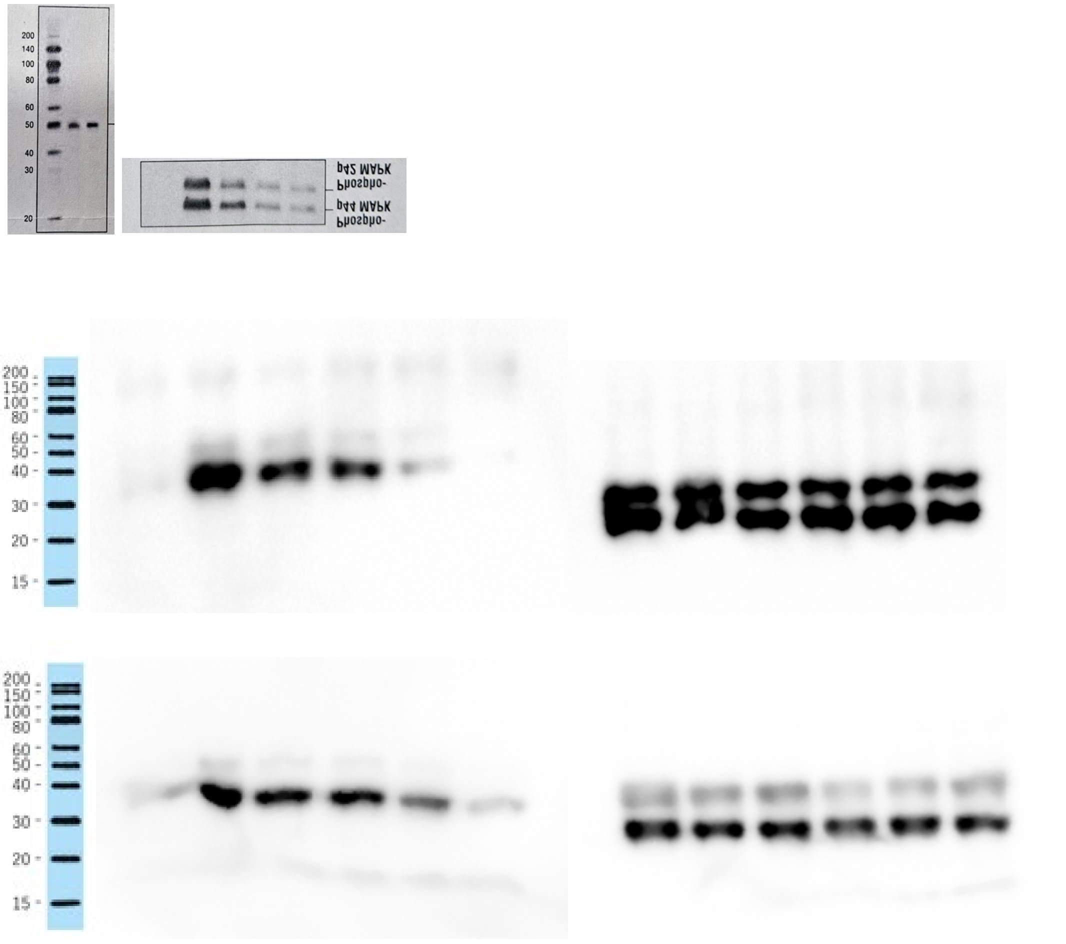

Supplementary Figure S6b

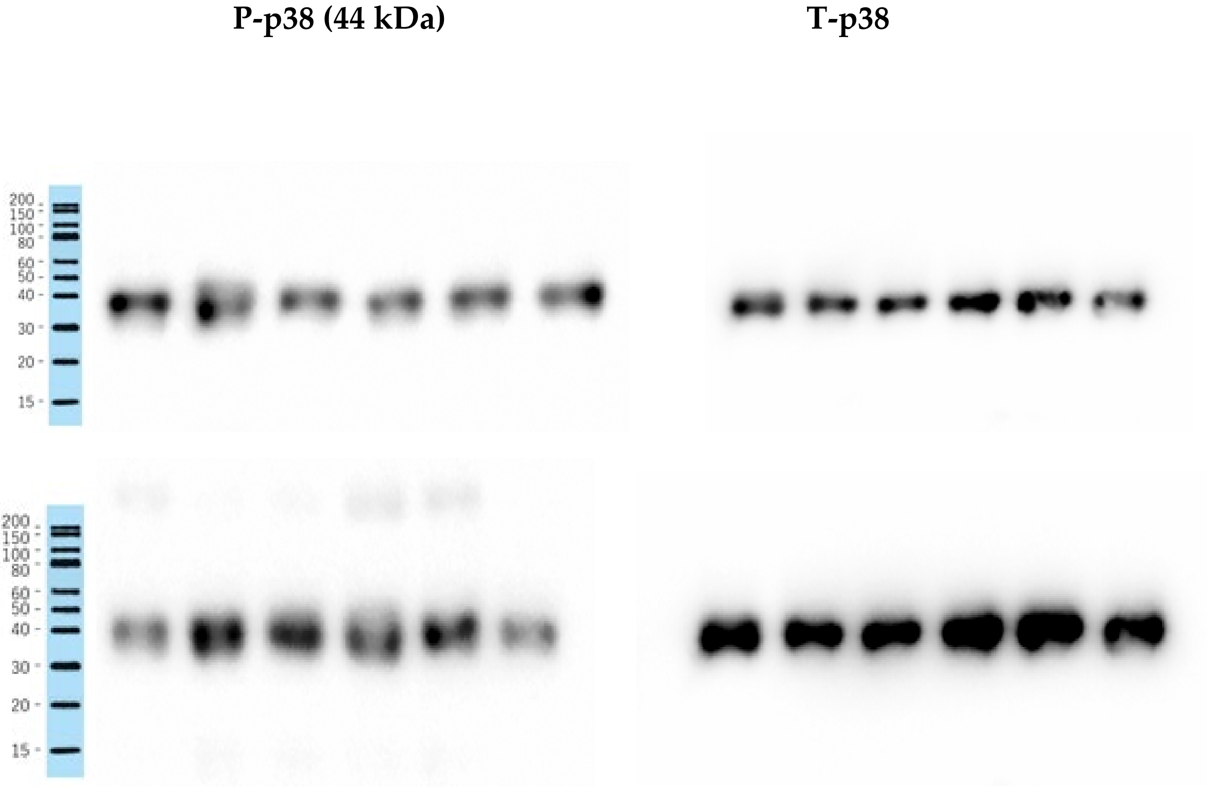

Supplementary Figure S6c

P-JNK (46-54 kDa) **T-JNK**

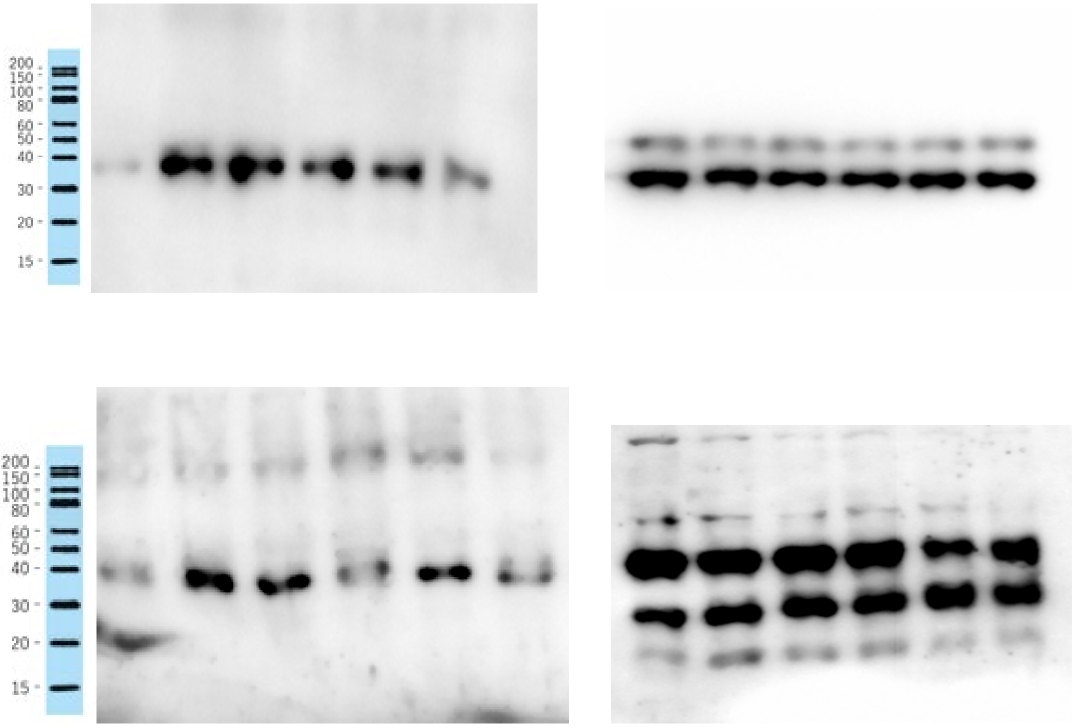

Supplementary Figure S6d

P-Akt (60 kDa) **T-Akt**

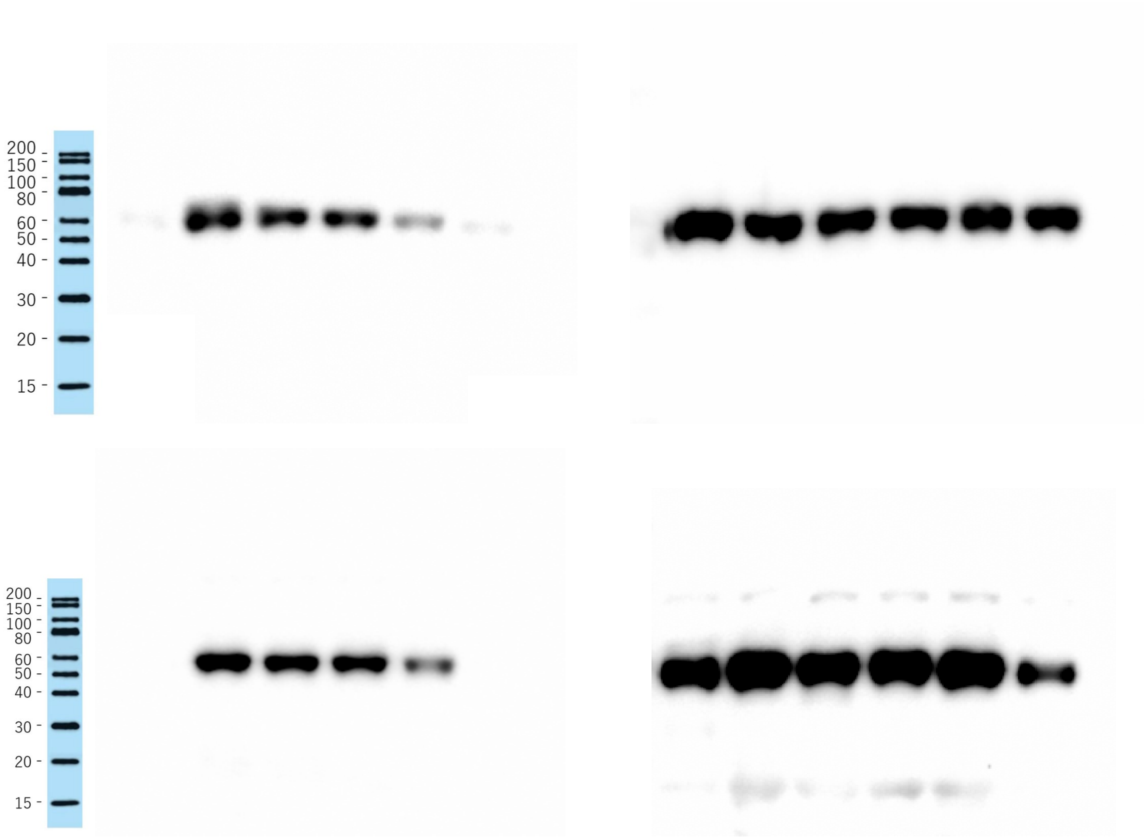

Supplementary Figure S6e

P-PI3K (85 kDa) **T-PI3K**

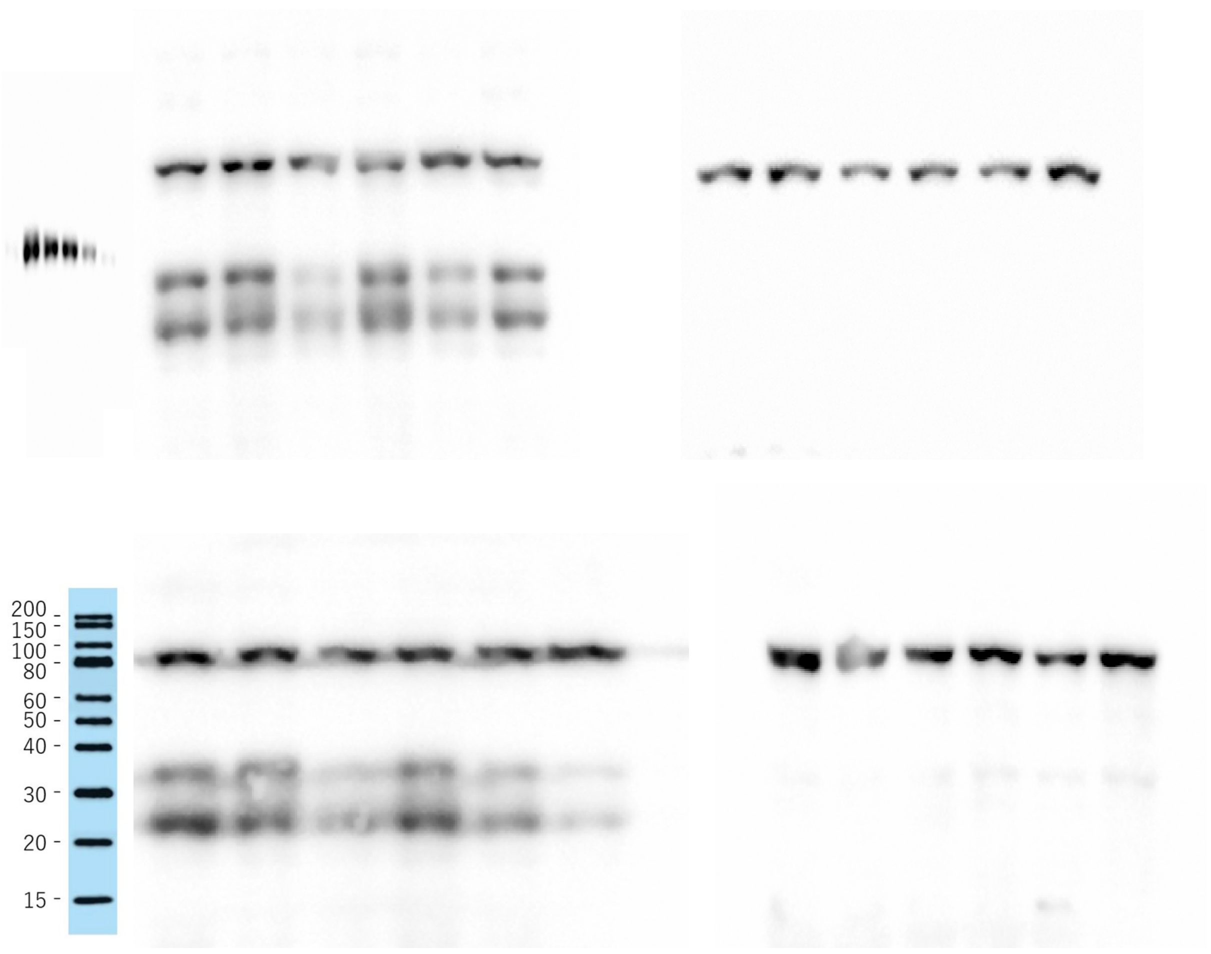

1.3 LDH cytotoxicity assay

DHG was examined for any cytotoxic effects via lactate dehydrogenase (LDH) leakage from the cytosol of platelets. Washed rat platelets were incubated with different concentrations of DHG for 2 hours and centrifuged for 2 min at 12,000 g. The supernatant was used to detect the cytotoxic effects using an LDH assay kit. The plate was read at a wavelength of 490 nm using Synergy HT Microplate Reader (BioTek Instruments, Winoosku, VT., USA).

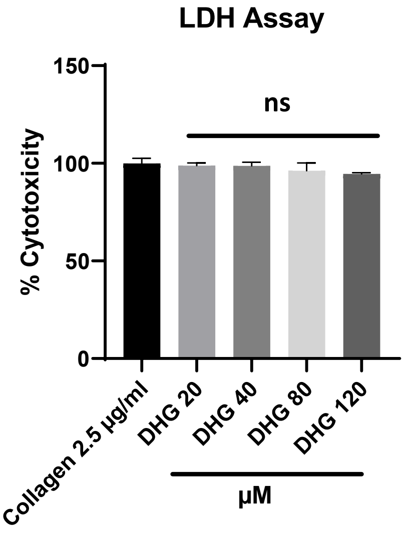

Figure S7. No potential cytotoxicity was observed with DHG. LDH assay was performed to evaluate the potential cytotoxic effects of DHG on rat platelets. DHG at 20-120 µM concentrations did not exhibit any potential cytotoxic effects to rat platelets. Here, statistical significance was determined by one-way ANOVA followed by a post hoc Dunnett's test, non-significant (ns) against collagen.
